# Proline affects the size of the root meristematic zone in Arabidopsis

**DOI:** 10.1186/s12870-015-0637-8

**Published:** 2015-10-29

**Authors:** Marco Biancucci, Roberto Mattioli, Laila Moubayidin, Sabrina Sabatini, Paolo Costantino, Maurizio Trovato

**Affiliations:** Dipartimento di Biologia e Biotecnologie, Sapienza, Università di Roma, P.le Aldo Moro 5, 00185 Rome, Italy

**Keywords:** Proline, Root meristem, Arabidopsis, *SHY2*, *CYB1;1*, Plant hormones, Cell cycle genes

## Abstract

**Background:**

We reported previously that root elongation in Arabidopsis is promoted by exogenous proline, raising the possibility that this amino acid may modulate root growth.

**Results:**

To evaluate this hypothesis we used a combination of genetic, pharmacological and molecular analyses, and showed that proline specifically affects root growth by modulating the size of the root meristem. The effects of proline on meristem size are parallel to, and independent from, hormonal pathways, and do not involve the expression of genes controlling cell differentiation at the transition zone. On the contrary, proline appears to control cell division in early stages of postembryonic root development, as shown by the expression of the G2/M-specific *CYCLINB1;1 (CYCB1;1*) gene*.*

**Conclusions:**

The overall data suggest that proline can modulate the size of root meristematic zone in Arabidopsis likely controlling cell division and, in turn, the ratio between cell division and cell differentiation.

**Electronic supplementary material:**

The online version of this article (doi:10.1186/s12870-015-0637-8) contains supplementary material, which is available to authorized users.

## Background

Thanks to its unique cyclic structure and physical-chemical properties, proline is of paramount importance in plants, both as building block for protein synthesis and as a compatible osmolyte accumulating during, and protecting from, environmental stress. It is synthesized in the cytosol from glutamate in a two-step pathway catalyzed by δ-pyrroline-5-carboxylate synthetase (P5CS), and δ-pyrroline-5-carboxylate reductase (P5CR). The first enzyme of this pathway, catalyzing the rate-limiting step of proline synthesis in higher plants, is encoded in Arabidopsis by two paralog genes *P5CS1* and *P5CS2,* while a single gene, *P5CR*, encodes the second committed enzyme of proline synthesis in plants [1].

In the last years it has been increasingly evident that the amino acid proline, in addition to its role in protein synthesis and stress response, plays a key role in plant development, particularly in developmental processes related to reproduction [2], such as flowering [3–6], pollen development [7, 8] and embryogenesis [2, 9].

Accordingly, Arabidopsis mutants carrying a knock out T-DNA insertion in *P5CS2* (FLAG_139H07, GABI_452G01) are embryo lethal in homozygosis, and can be propagated only as heterozygotes, unless complemented by exogenous proline [2, 9]. Furthermore, Arabidopsis mutants homozygous for *p5cs1* and heterozygous for *p5cs2* (*p5cs1 p5cs2/P5CS2*) are late flowering [2] and male sterile [7, 8]. A more general role as signal molecule involved in plant development, however, might also be assigned to proline on the basis of the claim that micromolar concentrations of exogenous proline promote root growth [6]. Intriguingly, the first indications of a role of proline in plant development, beyond protein synthesis and stress adaptation, came from the study of the adventitious roots induced by the soil bacterium *Agrobacterium rhizogenes* [10]. Virulent strains of this bacterium harbor a plasmid capable to transfer to, and integrate in the plant genome a portion of its own DNA, called T-DNA. The expression of some of the genes borne on this transferred DNA, notably *rolA*, *rolB*, *rolC* and *rolD*, are responsible of hairy root insurgence and elongation. This latter, by insertional mutagenesis [11], has been specifically attributed to *rolD*, later on recognized as a proline-producing ornithine cyclodeaminase gene [4], providing a direct correlation between proline availability and root growth. Moreover, proline was found, at low water potential, to accumulate preferentially in the root meristem growth zone of the maize primary root [12, 13].

In recent years our understanding of the genetic and molecular mechanisms underlying root growth and development has tremendously improved thanks to the exploiting of the model species *Arabidopsis thaliana*. The simplicity of its cellular organization, the possibility to be grown on agar plates under well-defined conditions, and the wealth of genetic and molecular resources available for Arabidopsis, have greatly contributed to build a solid picture of the molecular mechanisms behind growth and development of the Arabidopsis root [14]. The dimension of the root meristematic zone, which relies on the ratio between cell division in the meristem region, and cell differentiation in the transition zone (TZ), is pivotal for postembryonic root growth, and is regulated by the plant hormones auxin, cytokinin and gibberellin which, in turn, control a short regulatory circuit converging on the gene *SHY2* [15]*.*

According to the current model [15], *SHY2* is induced by the cytokinin-responsive transcription factors ARR1 and ARR12*,* and regulates the size of the root meristem by downregulating the *PIN-FORMED* (*PIN*) genes that encode auxin efflux facilitators. In addition to plant hormones, however, novel effectors have been recently proposed to affect root meristem size in Arabidopsis [16–18] and others are likely to be found, as plants, being sessile organisms, must be able to respond to a multiplicity of different stimuli. To test the hypothesis that proline may be one of such effectors, we used a combination of genetic, molecular and pharmacological analyses to study the growth of the primary root in proline-deficient mutant *p5cs1 p5cs2/P5CS2*, compared to wild type. Here we show that proline can modulate the size of root meristematic zone in Arabidopsis by controlling cell division and, in turn, by modulating the ratio between cell division and cell differentiation.

## Results

### Proline stimulates growth of the root meristematic zone

**Fig. 1:**
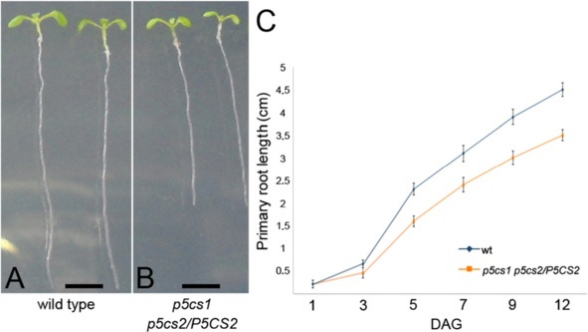
Proline specifically modulates root growth. **a-b** Roots from wild type (**a**) and *p5cs1 p5cs2/P5CS2* (**b**) grown on agar plates for 5 dag. **c** Primary root lengths of *p5cs1 p5cs2/P5CS2* (orange line) and wild type (blue line) plotted over time from 1 to 12 dag. The data are means ± SE of at least 90 samples from 3 independent experiments

**Fig. 2:**
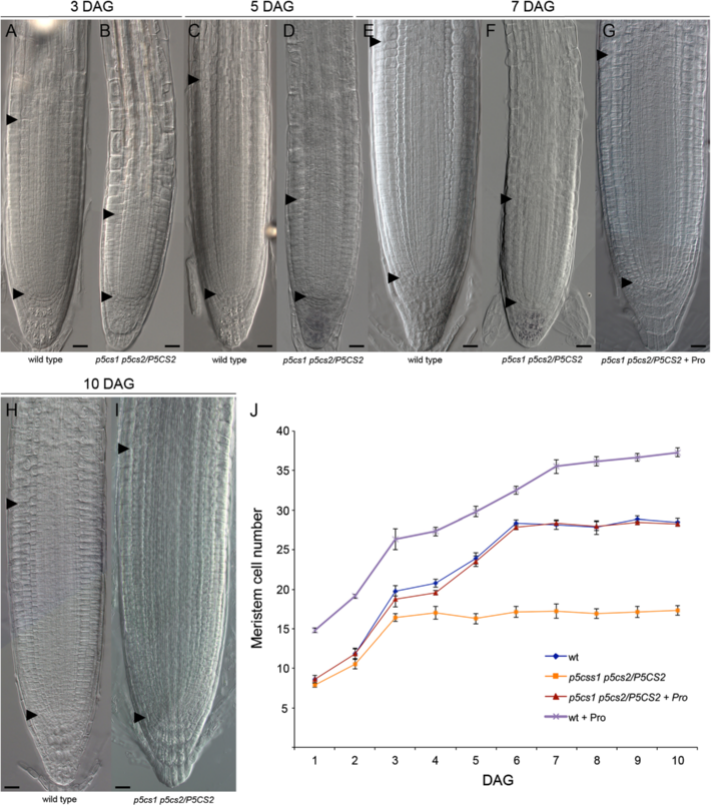
Proline-deficient mutants have meristems smaller than wild types. **a-f** Root meristems from wild type (**a, c, e**) and *p5cs1 p5cs2/P5CS2* (**b, d, f**), at 3 (**a, b**), 5 (**c, d**) and 7 dag (**e, f**). Bottom black arrowheads indicate the QC, top black arrowheads indicate the cortex TZ. **g** Root meristem from *p5cs1 p5cs2/P5CS2* treated, at 7 dag, with 10 μM exogenous proline. **h-i** Wild-type root, at 10 dag, treated with 10 μM exogenous proline (**i**) compared with an untreated control (**h**). Bars = 20 μm (**a-i**). **j** Root meristem cell number of plants described in (**a**) to (**i**) plotted over time from 1 to 10 dag. The data are the means ± SE of at least 3 independent experiments. A minimum of 50 roots per line was analyzed at each time point

**Fig. 3:**
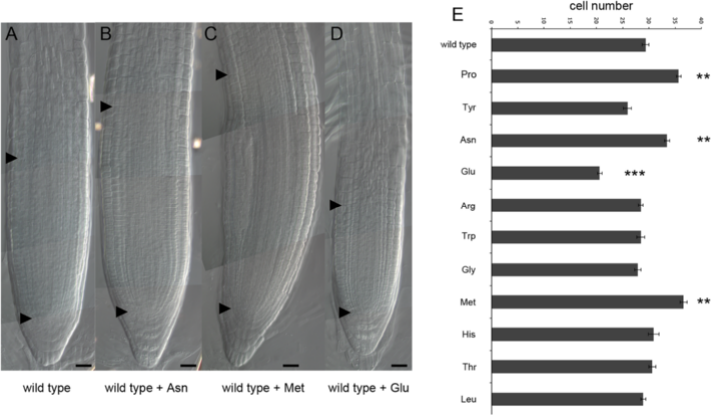
Effects of different amino acids on root meristem size. **a-d** Wild type roots at 7 dag treated with (**b**) 10 μM asparagine (Asp), (**c**) 10 μM methionine (Met), (**d**) 10 μM glutamate (Glu) compared to wild type (**a**). Bottom black arrowheads indicate the QC, top black arrowheads indicate the cortex TZ. Bars = 20 μm. **e** Bar plot showing the effects of 10 amino acids on the size of a wild-type root meristem. The amino acids were supplied *in vitro* at the concentration of 10 μM and the root meristem cells were scored at 7 dag. Apart from Asp, Met and Glu treatment, which led to meristems significantly larger (Asp, Met), or smaller (Glu) than untreated wild type meristems, all the other amino acids produced no effect on meristem size when supplied exogenously. Error bars indicate Standard Error (SE). The data are the means ± SE of at least 3 independent experiments. Significance levels for each amino acid treatment were calculated, relative to untreated controls, with a paired Student’s t-Test. p*** < 0.001; p** < 0.01

We reported previously that root elongation in Arabidopsis is promoted by micromolar concentrations of exogenous proline [2]. In order to verify whether a proline-deficient mutant is hampered in root growth, we analyzed the length, relative to wild type, of roots from the proline-deficient partial double mutant *p5cs1 p5cs2/P5CS2* [2] from 1 to 12 days after germination (dag). The proline content of this partial double mutant was measured at 7 and 14 dag in roots, confirming that this mutant contains, on average, one fourth as much proline as a wild type (0.050 ± 0.03 compared to 0.23 ± 0.02 μmoles/g (fresh weight), for proline-deficient mutants and wild types, respectively; *p* < 0.01). As shown in Fig. 1a to c, from 3 dag on, the roots of these mutants are shorter than wild type supporting the notion that proline stimulates root elongation. To further verify the correlation between proline and root growth, we analyzed the proline content and the length of roots from heterozygous *p5cs2/P5CS2* and homozygous *p5cs1* parental lines, compared to partial double mutant *p5cs1 p5cs2/P5CS2* and wild type lines*.* The proline content of the parental lines turned out to be intermediate between *p5cs1 p5cs2/P5CS2* and wild type lines, with measured values of 0.15 ± 0.05 μmoles/g of proline for homozygous *p5cs1* roots, and 0.11 ± 0.02 μmoles/g of proline for heterozygous *p5cs1/P5CS2* roots*.* In spite of the reduction in proline content, roots from homozygous *p5cs1* mutants appeared indistinguishable from wild type roots, while roots from heterozygous *p5cs1/P5CS2* looked slightly shorter (not shown) suggesting that the levels of endogenous proline present in these mutants are reduced, relative to wild type, but still sufficient (*p5cs1*) or nearly sufficient (*p5cs2/P5CS2*) to sustain normal root growth. Overall these data confirm the positive correlation between proline content and root growth. Clearly mutations on *P5CS2* have a stronger effect on proline accumulation and root growth, compared to mutations on *P5CS1*. However both genes seem to contribute to the overall proline content in roots, as indicated by the lower proline level and by the shorter roots exhibited by the *p5cs1 p5cs2/P5CS2* partial double mutant, compared to parental lines. In Arabidopsis, the maintenance of the root meristematic zone and, consequently, of the root growth is ensured by the balance between the rate of cell division in the root meristematic zone and the rate of cell differentiation in the TZ [15, 19]. To establish whether the reduction in root length of the proline-deficient mutant may derive from a reduction in meristem size, we measured, in *p5cs1 p5cs2/P5CS2* mutant and in wild type, the size of the root meristematic zone expressed as number of cortex cells spanning from the quiescent center (QC) to the first elongated cell in the TZ. As shown in Fig. 2, from 2a to 2g, and in Fig. 2j, the shorter roots of *p5cs1 p5cs2/P5CS2* are accounted for by smaller meristems that stop growing at 3 dag with an average number of cells of 16.4 ± 0.47 (Fig. 2j). The wild-type meristem, by contrast, reaches the balance between dividing and differentiating cells between 5 and 6 dag (Fig. 2j) with an average number of cells of 28.3 ± 0.33 (*p* < 0.001; wild type v/s *p5cs1 p5cs2/P5CS2*). To confirm the effect of proline on the size of the root meristematic zone, we scored the number of meristem cells in wild-type roots, grown either in presence or in absence of proline (Fig. 2j), at the optimal concentration of 10 μM as inferred by the dose-response curves shown in Additional file 1: Figure S1. Proline treatment (Fig. 2j) significantly (*p* < 0.001) increased meristem size from 1 to 10 dag, with an average number of 37.3 ± 0.54 cells, as compared to 28.5 ± 0.42 of the untreated controls (Fig. 2h and i). More importantly, the addition of exogenous proline was able to fully complement the reduction in meristem size of *p5cs1 p5cs2/P5CS2* roots (Fig. 2g and j). Moreover, in the meristem cells of homozygous *p5cs1* and heterozygous *p5cs2/P5CS2* roots, we scored an average number of 28.1 ± 0.41 and 25.5 ± 0.21 cells, respectively, in good correlation with their measured proline levels of 0.15 ± 0.05 and 0.094 ± 0.08 μmoles/g. To further investigate the specificity of proline to modulate meristem size and to rule out a generic stimulatory effect of amino acids as a source of supplemental nitrogen, we analyzed, in 7-days-old wild-type roots, the effects of different amino acids on the size of the root meristem. Wild-type seedlings were grown on different Petri dishes, each one supplemented with one of the amino acids shown in Fig. 3e, at the concentration of 10 μM. As shown in Fig. 3 (a to e), most of the tested amino acids (tyrosine, arginine, tryptophan, glycine, histidine, threonine and leucine) had no significant effect on the size of the root meristem size. Two amino acids (methionine, asparagine), however, had stimulatory effects on root meristem size (Fig. 3b, c and e), and one amino acid (glutamic acid) caused a reduction of root meristem size (Fig. 3d and e). Overall, these experiments indicate that amino acids do not have *per se* a generic stimulatory effect on meristem size, and that proline and few others amino acids may have a special role as metabolic or signaling molecule. To additionally validate the specific effects of proline on root growth, we analyzed, at 7 dag, the total protein profile of root tips (Additional file 2: Lane 2 and 4) of either wild type or *p5cs1 p5cs2/P5CS2* plants. As shown in supplemental Fig. 2 we found no significant difference in the accumulation of total proteins between wild type and *p5cs1 p5cs2/P5CS2* mutants indicating that the difference in root length and root meristem size between *p5cs1 p5cs2/P5CS2* and wild type are not caused by gross variations in protein accumulation.

### The effect of proline on root meristem size is independent from hormone action

**Fig. 4:**
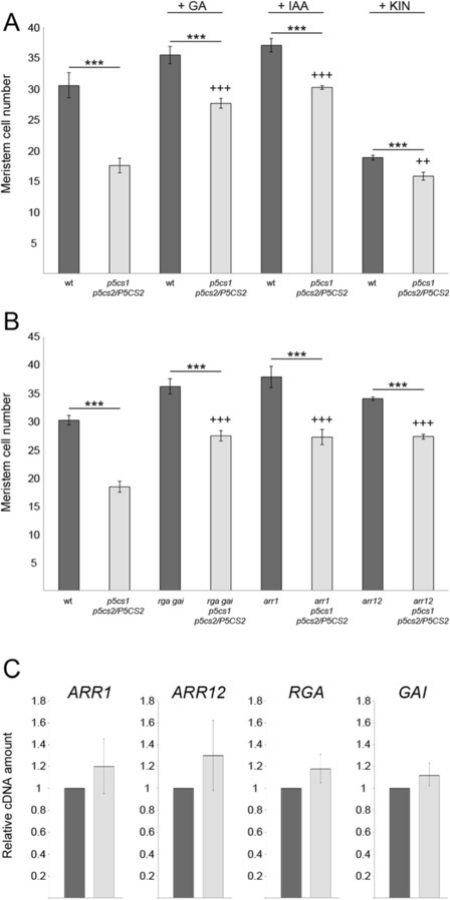
Proline effects on meristem size are independent from GA, IAA and cytokinin. **a** Root meristem sizes, measured at 7 dag as number or cortex cells spanning from the QC to the TZ, of wild types (dark grey bars) and *p5cs1 p5cs2/P5CS2* (light grey bars), upon pharmacological treatment with either 20 μM GA3, 0.1 nM IAA, or 10 μM kinetin. Error bars indicate Standard Error (SE). All pairwise comparisons (Student’s t test) showed that proline mutants treated with either GA3- or IAA, had root meristems highly significantly larger than untreated mutants (*p* < 0.001 +++), and highly significantly smaller than treated wild types (*p* < 0.001***). Kinetin-treated proline mutants, however, had root meristems highly significantly smaller than either treated mutants (*p* < 0.001***) , but only significantly smaller than untreated wild types (*p* < 0.01 ++). **b** Root meristem sizes, at 7 dag, of genetic combinations (light grey bars) mimicking either GA3 treatment (*p5cs1 p5cs2/P5CS2, gai-t6, rga-24),* or kinetin treatment (either *p5cs1 p5cs2/P5CS2, arr1* and *p5cs1 p5cs2/P5CS2, arr12*), compared to parental lines (dark grey bars). **c** qRT-PCR analysis shows no statistically significant differences in the expression levels of *ARR*1, *ARR12*, *GAI* and *RGA* between *p5cs1 p5cs2/*P5CS2 (light grey) and wild-type controls (dark grey). *ACTIN 8* was used as a reference gene to normalize the qRT-PCRs

Since root growth is regulated by the combined action of auxin, cytokinin and gibberellin, we searched for possible interactions between proline and these hormones. As a first approach, we analyzed the size of the root meristematic zone of *p5cs1 p5cs2/P5CS2*, compared to wild-type plants, upon exogenous treatment with either gibberellin (GA), indol-3-acetic acid (IAA) or cytokinin. As described previously [15, 19], supplementation of either GA or IAA to wild-type roots results in larger meristems, while cytokinin produces smaller meristems (Fig. 4a). After treatment with either GA or IAA the root meristematic zone of *p5cs1 p5cs2/P5CS2* appears significantly larger than untreated controls (*p* <0.001), but significantly smaller than hormone-treated wild types (*p* <0.001; Fig. 4a), indicating that the effects of auxin and gibberellin on root growth are antagonistic to those of proline deficiency. To confirm the results obtained with GA treatment, we crossed *p5cs1 p5cs2/P5CS2* with *gai-t6, rga-24* [20] *-* a double mutant line exhibiting constitutive GA response*.* As shown in Fig. 4b, *p5cs1 p5cs2/P5CS2, gai-t6, rga-24* displays a number of root meristem cells intermediate between *p5cs1 p5cs2/P5CS2* and *gai-t6, rga-24,* consistent with the pharmacological results described above. Moreover, the expression levels of either *GAI* or *RGA,* two master regulators of GA response*,* are similar in *p5cs1 p5cs2/P5CS2* and wild type, as shown in Fig. 4c, further suggesting that proline does not affect the GA pathway. In contrast, cytokinin-treated *p5cs1 p5cs2/P5CS2* meristems were smaller than both untreated and treated controls, indicating that the effects of cytokinin on root growth are additive to those of proline deficiency. To confirm these data at genetic level, we crossed *p5cs1 p5cs2/P5CS2* with either *arr1* or *arr12 -* two mutant lines defective in *ARR1* or, respectively *ARR12* - two genes coding for positive regulators of cytokinin response. As previously described [19] and shown in Fig. 4b, both *arr1* or *arr12* null mutants have root meristems larger than wild types. Once again, both *p5cs1 p5cs2/P5CS2, arr1* and *p5cs1 p5cs2/P5CS2, arr12* show a number of cells intermediate between the respective parental lines (Fig. 4b). In addition, the expression level of both *ARR1* and *ARR12* are similar in *p5cs1 p5cs2/P5CS2* and wild type, as judged by qRT-PCR (Fig. 4c), indicating that proline does not affect the key regulators of the cytokinin pathway. In conclusion, the combination of pharmacological, genetic and molecular data shows that the effect of proline on root meristem size is synergistic to IAA and GA and antagonistic to cytokinin and suggests that proline acts parallel to, and independent from hormonal pathways.

### Proline does not affect the activity of SHY2

According to the current model, *SHY2* [21] is induced by the cytokinin-responsive transcription factors ARR1 and ARR12*,* and plays a major role in root meristem size determination by downregulating the *PIN* genes that encode auxin efflux facilitators [15]. Based on the above-described results, if proline action is independent of cytokinin, *SHY2* expression should not be affected in the *p5cs1 p5cs2/P5CS2* background. However we could not rule out the possibility that *SHY2* itself could be a direct or indirect target of proline action. To assess this point we analyzed the expression of either *SHY2* or *SHY2::GUS* in a *p5cs1 P5cs2/P5CS2* background. As shown in Additional file 3: Figure S3A, the level of *SHY2* expression, from 1 to 7 dag, was essentially the same in wild type and *p5cs1 P5cs2/P5CS2* plants*,* either treated or non-treated with 10 μM proline, confirming that proline does not interact with cytokinin signaling nor directly affects *SHY2* expression. To strengthen this evidence, we analyzed the expression of *SHY2::GUS* driven by the promoter of *SHY2* [22], in wild type (Additional file 3: Figure S3B, S3F, S3J, S3N), *p5cs1 P5cs2/P5CS2* (Additional file 3: Figure S3C, S3G, S3K, S3O), proline-treated wild type (Additional file 3: Figure S3D, S3H, S3L, S3P), and proline-treated *p5cs1 P5cs2/P5CS2* plants (Additional file 3: Figure S3E, S3I, S2M, S2Q). We examined the expression of *SHY2::GUS* at 1 (Additional file 3: Figure S3B-E), 3 (Additional file 3: Figure S3F-I), 5 (Additional file 3: Figure S3J-M) and 7 (Additional file 3: Figure S3N-Q) dag, and never observed a significant difference in GUS expression, supporting the notion that *SHY2* expression is unrelated to proline content. Moreover, it should be pointed out that despite the lack of significant differences in *SHY2::GUS* expression between proline-treated and proline-untreated roots, the meristems of the former are larger than those of the latter (Compare Additional file 3: Figure S3H, S3L and S3P with Figure S3F, S3J and S3N and Figure S3I, S3M and S3Q with Figure S3G, S3K and S3O), corroborating the notion that proline stimulates root-meristem growth without influencing transcription of *SHY2.* To rule out the possibility that proline may act post-transcriptionally on the activity of the SHY2 protein, we analyzed by qRT-PCR the expression of *PIN1*, which is directly downregulated by SHY2 [19], in the *p5cs1 p5cs2/P5CS2* mutant and in the wild type. As shown in Additional file 3: Figure S3R, the expression level of *PIN1*, at 3 and 5 dag, is similar in *p5cs1 p5cs2/P5CS2* and wild-type roots, suggesting that proline does not affect the activity of the SHY2 protein.

### Proline affects the expression of *CYCB1;1* in the root meristematic zone

**Fig. 5:**
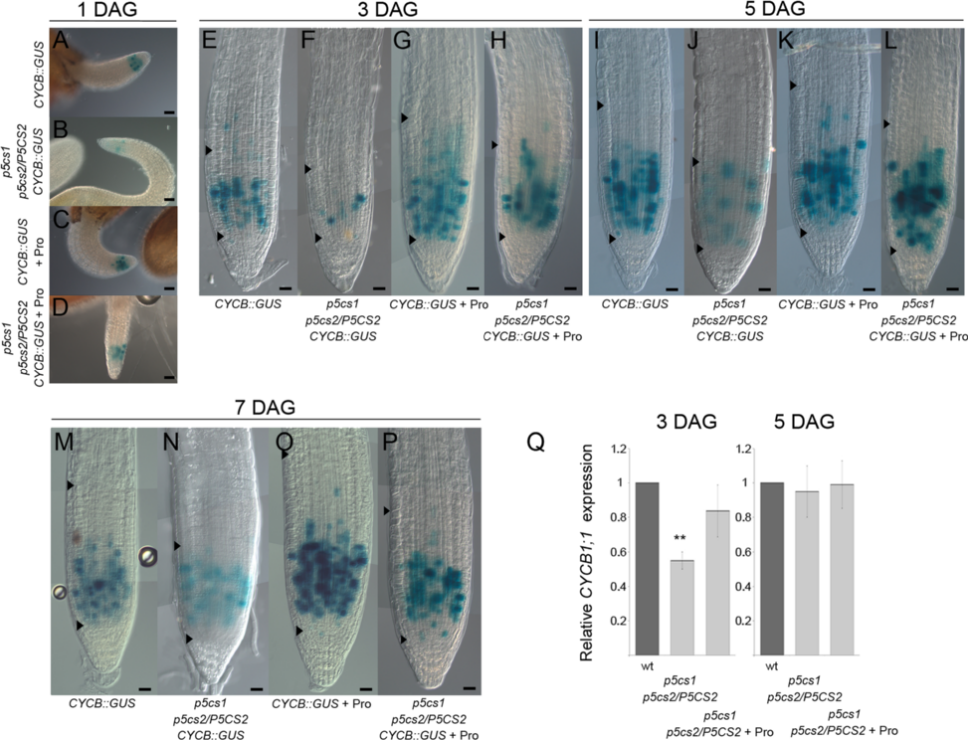
Proline affects the expression of *CYCB1;1* in the root meristematic zone. **a-p** *CYCB1::GUS* expression, from 1 to 7 dag, in roots from *CYCB1::GUS* (**a, e, i, m**), *p5cs1 p5cs2/P5CS2, CYCB1::GUS* (**b, f, j, n**), proline-treated *CYCB1::GUS* (**c, g, k, q**) and proline-treated *p5cs1 p5cs2/P5CS2, CYCB1::GUS* (**d, h, l, p**). Bottom and top arrowheads show meristem size indicating the QC and, respectively, the TZ. Bars = 50 μm (**a-d**), 20 μm (**e-p**). **q** qRT-PCR of *CYCB1;1*, at 3 and 5 dag, in root meristems of wild type (dark grey bar), *p5cs1 p5cs2/P5CS2* (light grey bar), and proline-treated *p5cs1 p5cs2/P5CS2* (grey bar), showing, at 3 dag, a strong downregulation of *CYB1;1* expression in *p5cs1 p5cs2/P5CS2* roots. The meristem-specific gene *RCH1* was used as reference control to normalize the qRT-PCR

To assess whether proline can modulate the size of the root meristematic zone by controlling cell division, we analyzed by RT-PCR (not shown) and qRT-PCR (Fig. 5q), the expression of *CYCLIN B1;1* (*CYCB1;1) -* a G2/M phase-specific cyclin gene regarded as a reliable marker of cell cycle progression [23] - in *p5cs1, p5cs2/P5CS2,* either treated or non-treated with exogenous proline*,* as compared as to a wild-type root meristem. Since *CYCB1;1* is expressed only in meristem cells, unlike the other genes analyzed in this work, we used the meristem-specific *ROOT CLAVATA HOMOLOG 1* (*RCH1*) [24] as a reference gene to normalize *CYCB1;1* expression to root meristems of different sizes. As shown in Fig. 5q, *CYCB1;1* is downregulated at 3 dag, but not at 5 dag, when the level of expression of this gene, relative to *RCH1*, becomes similar in the proline-deficient mutant and in the wild type. Supplementation of 10 μM exogenous proline to *p5cs1, p5cs2/P5CS2* roots, however, restored the levels of *CYCB1;1* expression to wild-type levels, confirming the effect of proline on the expression of *CYCB1;1*.

To further substantiate this evidence, we introgressed a *CYCB1::GUS* construct in *p5cs1, p5cs2/P5CS2*, and analyzed the activity of *CYCB1::GUS* in a *p5cs1, p5cs2/P5CS2 (*Fig. 5b, d, f, h, j, l, n, p), and wild type background (Fig. 5a, c, e, g, i, k, m, q), either without (Fig. 5a-b, e-f, i-j, m-n) or with (Fig. 5c-d, g-h, k-l, q-p) proline induction. As judged by the number of blue spots visible in Fig. 5, at 1, 3, 5 and 7 dag, respectively, the expression of *CYCB1::GUS* appears downregulated in the proline-deficient mutant *p5cs1, p5cs2/P5CS2* (Fig. 5b, f, j, n)*,* compared to wild type (Fig. 5a, e, i, m). After proline treatment, in contrast, the expression of *CYCB1::GUS* appears upregulated in a wild-type background (Fig. 5c, g, k, o), as well as in a *p5cs1, p5cs2/P5CS2* background (Fig. 5d, h, l, p), with the expression of *CYCB1::GUS* rescued to the levels of an untreated wild type. Overall these data show a positive correlation between proline content and cell cycle activity in the root meristematic zone.

The expression levels of *CYCB1;1* in *p5cs1, p5cs2/P5CS2* plants, as inferred by GUS staining and qRT-PCR analysis, appear somehow conflicting at 5 dag, since very few blue spots are visible in *p5cs1, p5cs2/P5CS2 CYCB1;1,* while the relative levels *of CYCB;1* transcripts are similar in *p5cs1, p5cs2/P5CS2* and wild type plants. The apparent conflict between GUS and qRT-PCR data at 5 dag arises because GUS analysis measures *CYCB1;1* expression in root meristem, while qRT-PCR measures *CYCB1;1* expression per root meristem, as ratio of *CYCB1;1* over *RCH1* expression. Indeed, by normalizing the number of GUS spots to the meristem size , i.e. plotting the ratio between the number of GUS spots and the number of cortex cells spanning from the QC to the TZ (Additional file 4: Figure S4), it became apparent that, at 1 dag this ratio is much lower in *p5cs1 p5cs2/P5CS2* than in wild type, while remains constant in time both in proline-untreated and in proline-treated wild-type roots. From 1 to 3 dag this ratio increases steadily in the mutant until it stabilizes at a value slightly lower than in the wild type, consistent with qRT-PCR data.

Since we never saw differences in germination rates between *p5cs1 p5cs2/P5CS2* and wild type seedlings (49.5 % ± 5 % for wt compared to 51.2 % ± 7 % for *p5cs1 p5cs2/P5CS2,* at 24h), the lower ratio of GUS spots to root meristem cells found in early stages of meristem growth of *p5cs1 p5cs2/P5CS2, CYCB1;1::GUS* roots, relative to wild-type *CYCB1;1::GUS* roots*,* indicates that proline-deficient meristems are growing at a slower pace than wild-type ones.

To further confirm this indication, we determined the rate of cell division in mutant and wild-type roots by calculating, with a modification of the Beemster and Baskin method [25], the variation of the number of root meristem cells over time. In agreement with qRT-PCR and GUS data, we found that in the first days of post-embryonic development root meristem cells of *p5cs1 p5cs2/P5CS2* grew slower than root meristem cells of wild types, with a rate of cell division at 3 dag of 0.018 ± 0.001 cells cells^−1^ h^−1^, compared to 0.022 ± 0.001 cells cells^−1^ h^−1^. At 5 dag the rate of cell division in the mutant root meristem dropped down to 0.008 ± 0.001 cells cells^−1^ h^−1^, in sharp contrast to the wild type root that showed a cell division rate of 0.012 ± 0.001 cells cells^−1^ h^−1^. The overall data indicate that in the root meristem of *p5cs1 p5cs2/P5CS2*, because of a slower cell division rate, the balance between cell division and cell differentiation is reached at 3 dag, when the root meristem of *p5cs1 p5cs2/P5CS2* gets its final dimension.

Upon proline treatment *CYCB1* is upregulated and cell division prevails over cell differentiation, consequently the meristem enlarges. However, since both blue-stained spots and meristem cells increase, the ratio between GUS-expressing and meristem cells remains unchanged in the proline-treated wild type. Our data are compatible with a model in which proline affects the ratio between cell division and cell differentiation, modulating, in turn, the size of the root meristematic zone. In the proline-deficient mutant, SHY2-mediated differentiation activity is normal, but cell division at early stages of meristem growth is hampered as *CYCB1* is downregulated. As a consequence, the *p5cs1 p5cs2/P5CS2* root meristem grows less in the first days after germination, and results in a smaller meristem than wild type, as the balance between cell division and cell differentiation is reached earlier (3 dag) than in the wild type (5 dag). To test this model we analyzed the effect of proline on meristem size upon variation of the expression of SHY2. We examined the root meristem size in a loss-of-function *shy2-31* mutant line [26] crossed with *p5cs1 p5cs2/P5CS2,* and in a *shy2-2* gain-of-function mutant line [27] treated with exogenous proline*.* Because of the absence of SHY2, the root meristematic zone of *shy2-31* null mutants never reaches a balance between cell division and cell differentiation and become much larger than in wild types.

**Fig. 6:**
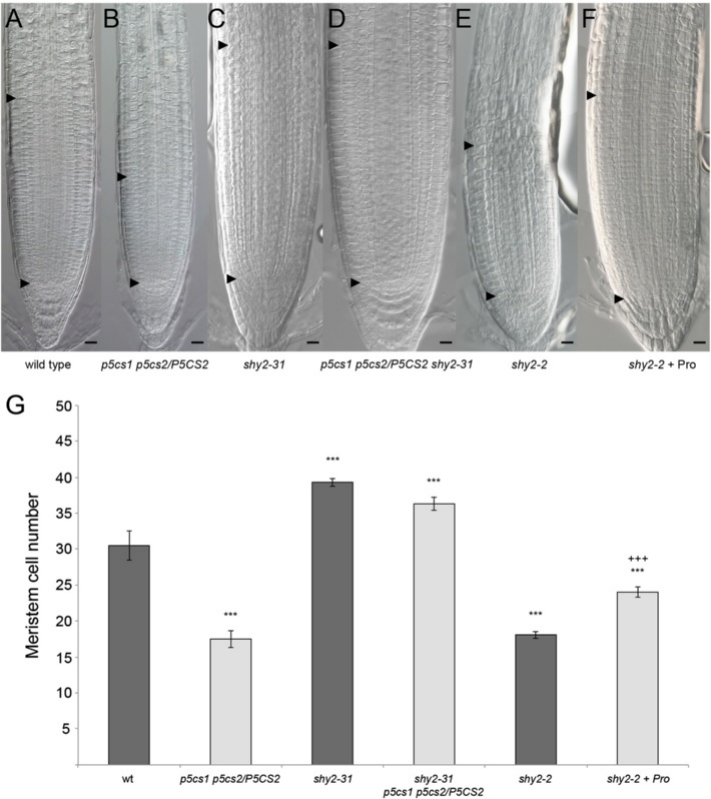
Effects of proline on *SHY2* loss- and gain-of-function mutants. **a-f** Longitudinal sections of roots, at 7 dag, from wild-type (**a**), *p5cs1 p5cs2/P5CS2* (**b**), *shy2-31* (**c**), *p5cs1 p5cs2/P5CS2, shy2-31* (**d**), *shy2-2* (**e**), *shy2-2* + proline (**f**), showing that in *p5cs1 p5cs2/P5CS2, shy2-31* the root meristem is as large as the meristem of *shy2-31*, and in proline-treated *shy2-2* larger than in untreated control. Black arrowheads indicate the QC (bottom arrowhead) and the TZ (top arrowhead). Bars = 20 μm (**a-f**). **g** The number of root meristem cells of the mutant lines shown above, from **a** to **f**, are reported as graphic bars. Error bars indicate Standard Error (SE). Student’s t test for wild type v/s mutant lines p*** < 0.001; Student’s t test for *shy2-2* v/s *shy2-2* + Pro p*** < 0.001

Consistently with the model, the root meristem of *p5cs1 p5cs2/P5CS2 shy2-31* was found to be much larger than the root meristem of *p5cs1 p5cs2/P5CS2* and nearly as large as the root meristem of *shy2-31* (Fig. 6c to d and g). The results on *shy2-2* upon proline treatment also support our model. *Shy2-2* is a gain-of-function mutant displaying a short root and a reduced root meristematic zone. If our model holds true we can anticipate that in spite of the high levels of SHY2 that accumulate in the *shy2-2* background, proline supplementation should move the boundary between cell division and differentiation resulting in larger meristems. Indeed, the root meristematic zones of proline-treated *shy2-2* were much larger than those of untreated controls (Fig. 6e, f and g). In conclusion, we point to proline as a novel effector capable of modulating the size of the root meristematic zone in Arabidopsis by modulating the ratio between cell division and cell differentiation.

## Discussion

### Proline stimulates root elongation

The cyclic amino acid proline has been implicated in root elongation ever since the discovery of *rolD*, a gene from *Agrobacteriun rhizogenes* necessary for hairy roots elongation [11], and encoding an unusual ornithine cyclodeaminase that catalyzes the direct conversion of ornithine to proline [4]. Consistently, by a combination of GUS, qRT-PCR and kinematic analyses, we show that proline can affect root elongation in Arabidopsis by modulating the rate of cell division, expressed as cells cells^−1^ h^−1^ and calculated from the variation of the number of root meristem cells over time [25]. In Arabidopsis proline is mainly synthetized from glutamate. Two paralog genes, *P5CS1* and *P5CS2,* code for P5CS a bifunctional enzyme that catalyzes the rate-limiting conversion of glutamic acid to glutamic-γ-semialdehyde (GSA). Following spontaneous cyclization, GSA is converted in δ-pyrroline-5-carboxylate (P5C) that is further reduced to proline by the enzyme P5CR. Despite high sequence similarity and partially overlapping transcription pattern, these two paralog genes seem to play different, non-redundant functions in stress regulation and embryogenesis, because *p5cs1* homozygous mutants are hypersensitive to stress conditions [9], while *p5cs2* homozygous mutants are embryo lethal [2, 9]. In root growth, however, *P5CS1* and *P5CS2* may have partially redundant functions. Indeed, while *p5cs1* homozygous mutants have roots indistinguishable from wild type and *p5cs2/P5CS2* heterozygous mutants have roots only slightly shorter (not shown), roots from *p5cs1 p5cs2/P5CS2* partial double mutants are always much shorter than parental mutants (Fig. 1a to c). Here we show that the shorter roots of *p5cs1 p5cs2/P5CS2* are accounted for by smaller meristems, and that mutant roots can be brought to wild type length by exogenous proline treatment. As reported in this paper, the effect of proline on root meristem is specific and cannot be caused by any generic amino acid as a source of surplus carbon and nitrogen because, as shown in Fig. 3, most of the amino acids exogenously supplied *in vitro* had no effects either on root growth (Fig. 3) or on *CYCB1:GUS* expression (not shown). Intriguingly, asparagine and methionine were found to increase while glutamate was found to decrease root meristem size, raising the question whether or not these amino acids may share a common mechanism with proline. The inhibitory effect of exogenous glutamate on root growth seems quite different from the effect of proline. Unlike other amino acids that inhibit root growth at high concentration, including proline, glutamate is effective only at low concentrations and leads to inhibition of the primary root but also to proliferation of secondary roots [28]. Its mechanism of action is known to involve signal transduction via MEKK1 kinase [29] and probably perception by some member/s of the plant family of the glutamate-like receptor homologs (GLRs) [30]. Methionine has been recently shown to inhibit autophagy and promote growth through S-adenosylmethionine-responsive methylation of Protein Phosphatase 2A [31] and may behave as a sensor of nutritional state involved in non-nitrogen starvation. Asparagine, together with leucine and glutamine, are regarded as the main effectors involved in the activation of mTORC1 in mammal cells. Although not fully understood, the mechanism of amino acid sensing has been recently shown to occur at the lysosome where mTORC1 is regulated through an amino acid sensing cascade involving RAG GTPases, the Ragulator complex and the vacuolar H^+^-ATPase [32]. It is tempting to speculate that proline, together with asparagine and possibly other amino acids, may serve in plants as sensor of amino acid sufficiency or limitation.

Apart from the short-root phenotype, no major defects are seen in the general plant growth of *p5cs1 p5cs2/P5CS2* compared to wild types, as judged by germination rates, rosette leaf diameters (0.1 cm ± 0.003 cm for wt compared to 0.12 cm ± 0.005 cm for *p5cs1 p5cs2/P5CS2*, at 7 dag), rosette leaf area (0.0049 cm^2^ ± 0.00029 cm^2^ for wt vs 0.0046 cm^2^ ± 0.00028 cm^2^ for *p5cs1 p5cs2/P5CS2,* at 7 dag) and fresh weight (1.06 mg ± 0.078 mg for wt vs 1.10 mg ± 0.076 mg for *p5cs1 p5cs2/P5CS2,* at 7 dag). The root architecture is somehow less branched compared to wild type, suggesting that proline may also affect secondary root development. However the effects of proline on secondary roots appear late in development and we don’t know, at present, whether these effects are direct or indirect. In addition *p5cs1 p5cs2/P5CS2* partial double mutants exhibit also a slight delay in flowering [2] and a reduced fertility [8], but overall they look normal and very different from the homozygous *p5cs2* mutants described by Funck et al. [7] in Arabidopsis and by Wang et al. [33] in Maize. The most likely explanation for these discrepancies is that, unlike *p5cs1 p5cs2/P5CS2,* the two latter mutants carry homozygous mutations in *P5CS2*, which have been associated to embryo lethality and severe morphological defects [2, 7, 9].

### Relationships between proline and plant hormones

Plant hormones play pivotal roles in plant growth and development and four of them, auxin, cytokinin, gibberellins and brassinosteroids are essential for plant growth, with auxin, cytokinin and gibberellins mainly involved in cell division, and brassinosteroids in cell elongation [34]. Since proline affects the root meristematic zone by modulating the number of meristem cells, similarly to auxin, cytokinin and gibberellins, we focused on these latter hormones to assess epistatic relationships among proline and plant hormones. According to the current view, root growth is largely determined by the generation of auxin gradients and local auxin maxima that are essential for establishing and maintaining the root meristematic zone. These local auxin levels mainly depend on the expression of the *PIN* genes, which, in turn, are controlled by the levels of SHY2. In a regulatory circuitry, the crosstalk between auxin, cytokinin and gibberellin finely tunes the expression of *SHY2* in the root to fix the position of the root transition zone and determine the boundaries of the root meristematic zone. Some authors [35, 36], however, reported that auxin could also affect cell cycle by controlling the expression of cell cycle regulators. Himanen et al. [35], for example, showed that auxin induces *CYCB1;1* expression in secondary roots of Arabidopsis, while Mähönen et al. [36], have recently shown in Arabidopsis that the auxin-induced *PLETHORA* (*PLT*) genes define the location of developmental zones of the primary root, and affect the expression of cell cycle regulator genes, including *CYCB1;1.*

These findings suggest that the effects of proline on cell division are mediated by auxin. However, the combination of pharmacological, genetic and molecular data presented in this work indicate that the effects of proline deficiency on root meristem size are antagonistic to IAA and GA and additive to cytokinin, and suggest that proline does not participate to, or interact with any of these hormonal pathways. With respect to auxin, in particular, we provide evidence that auxin-induced genes such as *PIN1* (Additional file 3: Figure S3R) and *SCARECROW* (*SCR*; Additional file 5: Figure S5) are not altered in a *p5cs1 p5cs2/P5CS2* background, corroborating the notion that proline does not interfere with auxin signaling. In addition, proline supplementation can partially rescue the small meristem size of the gain-of-function *shy2-2* mutant (Fig. 6c, d, g), while auxin supplementation cannot [37]. Notwithstanding we cannot rule out that proline and auxin signaling may converge on the modulation of cell cycle genes, to integrate hormonal and nutritional inputs and adjust root growth to optimal rate.

### The expression and the activity of *SHY2* is not altered in proline-deficient mutants

The AUX/IAA cytokinin-induced *SHY2* gene [21] is a master regulator of root growth in Arabidopsis and plays a crucial role in the crosstalk between auxin, cytokinin and gibberellin to define the final size of the root meristematic zone [19]. In response to cytokinin signaling, the transcription factors ARR1 and ARR12, induce the expression of *SHY2*, which, in turn, inhibits the expression of the *PIN1/3/7* genes and, eventually, stabilizes the boundary between cell proliferation and cell differentiation to its final position [19]. Furthermore it has been shown that *SHY2* is necessary and sufficient to control root meristem size in Arabidopsis [37]. Although, on the basis of our data, the effects of proline on root meristem size seem independent from cytokinin and unrelated to *ARR1* and *ARR12*, we could not rule out the possibility that other B-type ARR genes might modulate SHY2 expression, or that *SHY2* itself could be a target of proline action. As shown in results, the data from SHY2::GUS staining and *SHY2* expression indicated that proline affects neither the promoter activity nor the mRNA abundance of *SHY2*. In addition we ruled out possible translational or post-translational effects on SHY2 activity or stability, by showing that the expression of *PIN1*, an auxin efflux facilitator directly downregulated by SHY2 [37], is indistinguishable between wild types and *p5cs1 p5cs2/P5CS2* mutants.

We decided to study this particular PIN, because among the others shown to be affected by cytokinin signaling (PIN3 and PIN7) [37] is the one showing the strongest effects.

### May proline behave as a signal molecule?

Since the effects of proline on root meristem appeared independent from plant hormones and unrelated to the regulation of the boundary between dividing and differentiating cells, we investigated the rate of cell division in wild-types and *p5cs1 p5cs2/P5CS2* roots by following the expression of the G2/M phase-specific *CYCB1;1* cyclin gene. Our data clearly show a positive correlation between proline and *CYCB1;1* expression and, in turn, cell division activity, although, at present, we don’t know the molecular mechanism by which proline can modulate the rate of cell division of the primary root. A hint to explain the effects of proline on cell cycle is given by Wang et al. [38] who showed in maize that proline plays a critical role in regulating both general protein synthesis and cell cycle, suggesting that the proline deficiency of *p5cs1 p5cs2/P5CS2* mutants may be a limiting factor both for protein synthesis and cell cycle progression. However, from the analysis of protein accumulation in roots and shoots that found no significant difference between mutant and wild-type lines (See Additional file 2: Figure S2), and from the observation that *p5cs1 p5cs2/P5CS2* partial double mutants shows no major growth defects, apart from having roots shorter than wild type roots, we consider unlikely that the growth defects of *p5cs1 p5cs2/P5CS2* may derive from gross variations in protein accumulation caused by limiting amount of intracellular proline. It is possible, however, that similarly to other nutrients, such as phosphate [39] and sugars [40], proline can also behave as a signaling molecule. In this regard, the effects of auxin and gibberellin on the growth of *p5cs1 p5cs2/P5CS2* roots are a valid argument against the possibility that the reduction in root growth observed in *p5cs1 p5cs2/P5CS2* mutants may be simply caused by the rate-limiting effects of proline shortage on protein synthesis, rather suggesting a signaling effect. Similarly, the large root meristems observed in *shy2-31 p5cs1 p5cs2/P5CS2,* confirm that, provided the repressive action of SHY2 is released, meristem size and, in turn, root length increases in *shy2-31 p5cs1 p5cs2/P5CS2* as much as in *shy2-31* mutants, suggesting again that the amount of proline present in *p5cs1 p5cs2/P5CS2* is not rate limiting for root growth.

Another argument in favor of a signaling function of proline is that, only low concentrations of proline have a promoting effect on root growth (Additional file 1: Figure S1), while higher concentrations inhibit root growth [2, 41]. The capacity of proline to promote growth al low concentrations and to inhibit growth at higher concentrations is also found in some sugars, a class of metabolites for which a signaling function has been clearly demonstrated [42]. Glucose, for example, can trigger growth stimulation, at low concentration, and growth repression, at high concentration [42]. The correlation between proline concentration and developmental responses, which would be expected if proline acted as a limiting factor for protein synthesis, is also lacking under stress conditions when proline levels increase dramatically but growth is severely reduced [43]. As another example, in *eskimo1* mutants, constitutive accumulation of proline leads to plants slightly smaller than wild types at 22 °C, but indistinguishable from wild types under cold stress [44]. The accumulation of proline under stress conditions seems therefore uncoupled to growth modulation but rather associated to stress resistance and different signaling pathways may be involved in stressed and unstressed conditions for proline perception and transduction. Accordingly proline accumulation has been shown to induce incompatible plant-pathogen interactions in Arabidopsis by triggering a salicylic acid-dependent hypersensitive response [45, 46]. Moreover also in human cells proline catabolism is clearly implicated in ROS signaling, which controls programmed cell death and apoptosis. Enhanced proline oxidation in human carcinoma cells generates apoptotic signals capable to trigger programmed cell death to control cancer cell proliferation [47, 48]. Proline-dependent apoptosis is mediated by the TRAIL death receptor pathway, which activates caspase-8 [48] and, in turn, various signaling pathways such as Mitogen-activated protein kinase (MEK) and Extracellular signal-Regulated Kinase (ERK) pathways [48]. In yeast and mammals cells, on the other hand, some amino acids are known to act as signal molecules capable to activate the TOR pathway a signal transduction pathway involved in coupling metabolic status to cell growth [49]. The TOR pathway is also active in plants and has been recently shown to be activated by glucose to modulate root growth through profound transcriptionally global rearrangements [50].

Although this paper cannot answer to the long-lasting question whether or not proline may behave as a signaling molecule and further work is obviously needed to address this issue, the data presented here are compatible with a signaling role of proline in the modulation of root growth.

## Conclusions

Here we show that proline can specifically modulate the size of the root meristem independently from plant hormones, likely controlling the ratio between cell division and cell differentiation.

## Methods

### Plant material, growth conditions and treatments

Wild-type and mutants *Arabidopsis thaliana* from Columbia-0 (Col-0) ecotype were mostly used in this work, with the exception of *shy2-31*, which was in Landsberg erecta (Ler). Since the effects of proline on root length and root meristem size have been preliminary tested either in Col-0 or in Ler, and have been found to be indistinguishable between the two ecotypes (not shown), we decided to use Col-0 as wild-type control in all the experiments.

*p5cs1 p5cs2/P5CS2*, *arr1-4*, *arr1-3, gai-t6 rga-24, pSHY2::GUS*, and *pCYCB1;1::GUS* were previously described [2, 15, 18, 19]. When genetic crosses were done from different ecotypes, parental wild types were isolated to be used as reference. All plants were grown in a growth chamber at 24/21 °C with light intensity of 300-μE · m-2 · s-1 under 16 h light and 8 h dark per day. Seeds were surface sterilized with a 2.5 % aqueous solution of INOV’chlore (Inov Chem) for 10 min and then rinsed four times with sterile water. After 5 days of cold treatment, *A. thaliana* seeds were plated on one-half MS (Murashige and Skoog medium, Duchefa) supplemented with sulfadiazine – to counter select *P5CS2/P5CS2* from the *p5cs2/P5CS2* heterozygous population - and grown in vertical position. In all genetic crosses using *p5cs1 p5cs2/P5CS2,* this mutant was used as a female, because of the male sterility of the *p5cs1 p5cs2* pollen grains [8]. Proline, Cytokinin, gibberellin, and auxin treatments were carried out as described in figure legends. GUS analysis was carried out as described [2].

### Proline analysis

Proline content was measured according to Bates [51], using L-proline as standard. The absorbance was read at 520 nm with a Varian Cary 50 spectrophotometer and the calculated proline values were expressed as μmoles/g fresh weight. Every measurement represents the average from more than 1 hundred 14 dag-old seedlings coming from four independent experiments.

### Plant crosses

In crosses using *p5cs1 p5cs2/P5CS2*, this partial double mutant was always used as a female. The F1 generation was allowed to self fertilize and the presence of the *p5cs2* mutant allele was assessed from the F2 generation, by sulfadiazine selection or by PCR genotyping of the sulfadiazine resistance gene.

### Root-length and meristem-size analysis

Root length was measured with IMAGE J software (http://rsb.info.nih.gov/ij). For each experiment, at least 90 plants, coming from three independent experiments, were analyzed. For meristem size analysis, roots were cleared with a 8:3:1 mixture of chloral hydrate:water:glycerol, mounted on a glass slide, and observed, under Nomarski optics, with an Axio Imager.A2 (Zeiss) light microscopy. The size of root meristems was performed by counting the number of cortex cells in a file extending from the QC to the first elongated cell in the TZ, as described [17].

### Determination of cell division rates in root meristems of primary roots

Cell division rates in root meristems were determined with a modification of the kinematic method described by Beemster and Baskin [25]. *A. thaliana* seeds were plated on one-half MS (Duchefa) supplemented with sulfadiazine and grown in vertical position. At daily intervals, roots were cleared with a 8:3:1 mixture of chloral hydrate:water:glycerol, observed under Nomarski optics with an Axio Imager.A2 (Zeiss) light microscopy and acquired with a DC500 digital camera (Leica, Germany). For every time point the average number of cortex cells (N) from a file extending from the QC to the TZ was determined. Next the number of dividing cells per root meristem was calculated by subtracting the number of cells from adjacent time points (N_2_-N_1_). Finally the rate of cell division, for every time point, expressed as cells cells^−1^ h^−1^, was calculated by dividing the resulting scores by N_1_*24 (N_2_-N_1_/N_1_* t_)_, and averaging the results from x data points. The difference in cell division rates between *p5cs1 p5cs2/P5CS2* and wild-type root meristem cells was tested for statistical significance with a student’s test a produced a significance level of **p < 0.05, at 3 dag, and ***p < 0.01, at 5 dag.

### Molecular techniques

Molecular techniques were performed according to standard protocols. Total RNA for RT-PCR was extracted from roots using RNeasy Plant Mini Kit (Quiagen) according to manufacturer’s instructions. Reverse transcription was performed from 1μg of total RNA using the Superscript II™ kit (Invitrogen) as recommended by the manufacturer. For genomic PCR *Arabidopsis* DNA was extracted with a modified CTAB method, according to Stewart and Via [52]. Primers and PCR conditions used for *p5cs1* and *p5cs2* were already described [2]. Primers for *ARR1, ARR12* and *ACT8* were as follows: ARR1-FW: 5′-GAGATGGCATTGTCTCTGCTC-3′; ARR1-RV: 5′-GATCAAACCCATT CAATGTCG-3′; ARR12-FW: 5′-CGGTACAATATGCGGATTTTGATTCGGTAT-3′; ARR12-RV: 5′-TCACCATTATTATTACTCCCACGGTTCTTA-3′; ACT-FW: 5′ -ATG AAGATTAAGGTC GTGGCA-3′; ACT-RV: 5′-TCCGAGTTTGAAGAGGCTAC-3′. PCR conditions were: 3′ at 94 °C followed by 30 cycles of 30″ at 94 °C, 30″ at 60 °C and 1′ at 72 °C. All primers used in this work were designed using Primer3 PLUS (http://www.bioinformatics.nl/cgi-bin/primer3plus/primer3plus.cgi/). Real-time qRT-PCR analyses were carried out with a Bio-Rad iCycler iQ (Bio-Rad). Amplifications were monitored using the SYBR Green fluorescent stain. The presence of a single PCR product was verified by dissociation analysis in all amplifications. The comparative threshold cycle (ΔΔC_T_) method was used to calculate the relative amount of gene expression, normalized using the C_T_ values derived for either *RCH1* (*CYCB1;1*) or *ACT* (*ARR1, ARR12, SHY2*). All the analyses were performed in triplicate on three independent samples. qRT-PCR Primers for *SHY2*, *ARR1, ARR12, CYCB1;1, RCH1* and *PIN1* were as follows: qSHY2-FW: 5′-AGATGGTGATTGGATGCTCA-3′; qSHY2-RV: 5′-GCCTAA ACCTTTGGCTTCTG-3′; qARR1-FW: 5′-TGGTACAGCACCATCAGGTT-3′; qARR1-RV: 5′-TGCTGCATC CGTAGCCACTC-3′; qARR12-FW: 5′-CTCTTCGACTCACCC TCCTC-3′; qARR12-RV: 5′-CACATTGTTCCATTCCAAGG-3′; qCYCB1 -FW: 5′-TGGTAGCTGCTTCTGCA ATC-3′; qCYCB1-RV: 5′-AGCTTTGCACAGTCCATGAG-3′; qRCH1-FW: 5′-AGAGAACGT GCCAAAGATGA-3′; qRCH1-RV: 5′-CGCAGAGAAA CTCGTGCTAC-3′; qPIN1-FW: 5′-GGTGGTGGTCGGAACTCTAA-3′; qPIN1-RV: 5′-TAGCAGGACCACCGTCT TCT-3′.

### Protein analysis

For protein analysis 5 mg (fresh weight) of root or shoot apexes from either wild type or *p5cs1 p5cs2/P5CS2* plants were collected. Roots were crushed in Laemmli buffer (1M Tris-HCl pH 6.8, 10 % SDS, 10 % glycerol, 5 % β-Mercaptoethanol, 0,04 % w/v BromophenolBue, 1:100 protease inhibitor cocktail (SIGMA-ALDRICH P9599) under liquid nitrogen and subsequently treated at 65 °C for 15 min. Equal volumes of each sample were loaded in a 30 % Sodium Dodecyl Sulphate PolyAcrylamide Gel (SDS-PAGE) for protein analysis. Protein detection was performed by Silver Staining according to Bio-Rad protocol (Silver Stain Plus™ Cat. N° #161-0449).

### Availability of supporting data

All the supporting data are included as additional files.

## Additional files

Additional file 1: Figure S1. Dose-response curve for proline-induced root elongation. Proline-induced elongation of Col-0 wild-type roots as a function of proline concentration, is shown, at 3 (upper panel) and 7 (bottom panel) dag. Proline concentration range from 0 (untreated control) to 100 μM. In both cases the maximum effects on root elongation occurs at 10 μM proline. All data are means of at least 20 roots ± SE. (PNG 194 kb) Additional file 2: Figure S2. Accumulation of protein in roots and shoots is no significantly different in mutant and wild-type lines. SDS-PAGE analysis of total proteins from shoot apexes (1,3) and root apexes (2,4) of wild type (1,2) and *p5cs1 p5cs2/P5CS2* plants (3,4). (PNG 587 kb) Additional file 3: Figure S3. Proline does not affect the expression of *SHY2*. (A) qRT-PCR of *SHY2* from 1 to 7 dag in wild-type (dark grey and grey bars) and *p5cs1, P5cs2/ P5CS2* (white and light grey bars) roots either untreated (dark grey and white bars) or treated (light grey and grey bars) with 10 μM proline. (B-Q) *SHY2::GUS* expression, from 1 to 7 dag, in a proline-treated *SHY2::GUS* (D, H, L, and P) compared to *SHY2::GUS* (A, F, J and N) and in a proline-treated *p5cs1 p5cs2/P5CS2, SHY2::GUS* (E, I, M and Q) compared to a *p5cs1 p5cs2/P5CS2, SHY2::GUS*. (C, G, K and O). Although the meristematic zone of roots from *p5cs1 p5cs2/P5CS2* is complemented to wild-type values by proline treatment, none of these lines exhibited significant differences in *SHY2* expression. Bars = 20 μm (B-Q). (R) qRT-PCR of *PIN1* at 3 and 5 dag in wild-type (dark grey bars) and *p5cs1, P5cs2/ P5CS2* roots. (PNG 3675 kb) Additional file 4: Figure S4. Ratio between *CYCB1;1::GUS* expression and the number of root meristem cells. Bar plot displaying the ratio between the number of GUS spots and the number of meristem cells, from 1 to 7 dag, in roots from *CYCB1::GUS* (dark grey bars), *p5cs1 p5cs2/P5CS2, CYCB1::GUS* (light grey bars), and proline-treated *CYCB1::GUS* (grey bars). Error bars indicate Standard Error (SE). *p* < 0.001***; *p** < 0.05 (Student’s t test). (PNG 129 kb) Additional file 5: Figure S5. Proline does not interfere with auxin signaling. (A-B) *SCR::GFP* expression in wild-type (A) and *p5cs1 p5cs2/P5CS2* roots (B), at 7 dag, show a similar pattern of expression under confocal microscopy. Bars = 20 μm. (PNG 499 kb)

## Abbreviations

*ACT8*: *actine 8*
ARR1: type-B Arabidopsis response regulator1
ARR12: type-B Arabidopsis response regulator12
AUX/IAA: auxin/indole-3-acetic acid
CTAB: cetyl trimethyl ammonium bromide
DNA: deoxyribonucleic acid
GAI: gibberellic acid insensitive
GUS: β-glucuronidase
MEKK1: mitogen-activated protein kinase kinase kinase 1
mTORC1: mammalian target of rapamycin complex 1
qRT-PCR: quantitative reverse transcription-polymerase chain reaction
RGA: repressor of *ga1-3*
*RolA*: *root locus A*
*RolB*: *root locus B*
*RolC*: *root locus C*
*RolD*: *root locus D*
ROS: reactive oxygen species
SDS: sodium dodecyl sulphate
*SHY2*: *short hypocotyl 2*
T-DNA: transferred DNA
TOR: target of rapamycin
Wt: wild type
