# Supplementary figures and images for: Proline affects the size of the root meristematic zone in Arabidopsis
Source: BMC Plant Biol. 2015 Oct 29;15:263. doi: 10.1186/s12870-015-0637-8 (PMC4625561; doi:10.1186/s12870-015-0637-8)

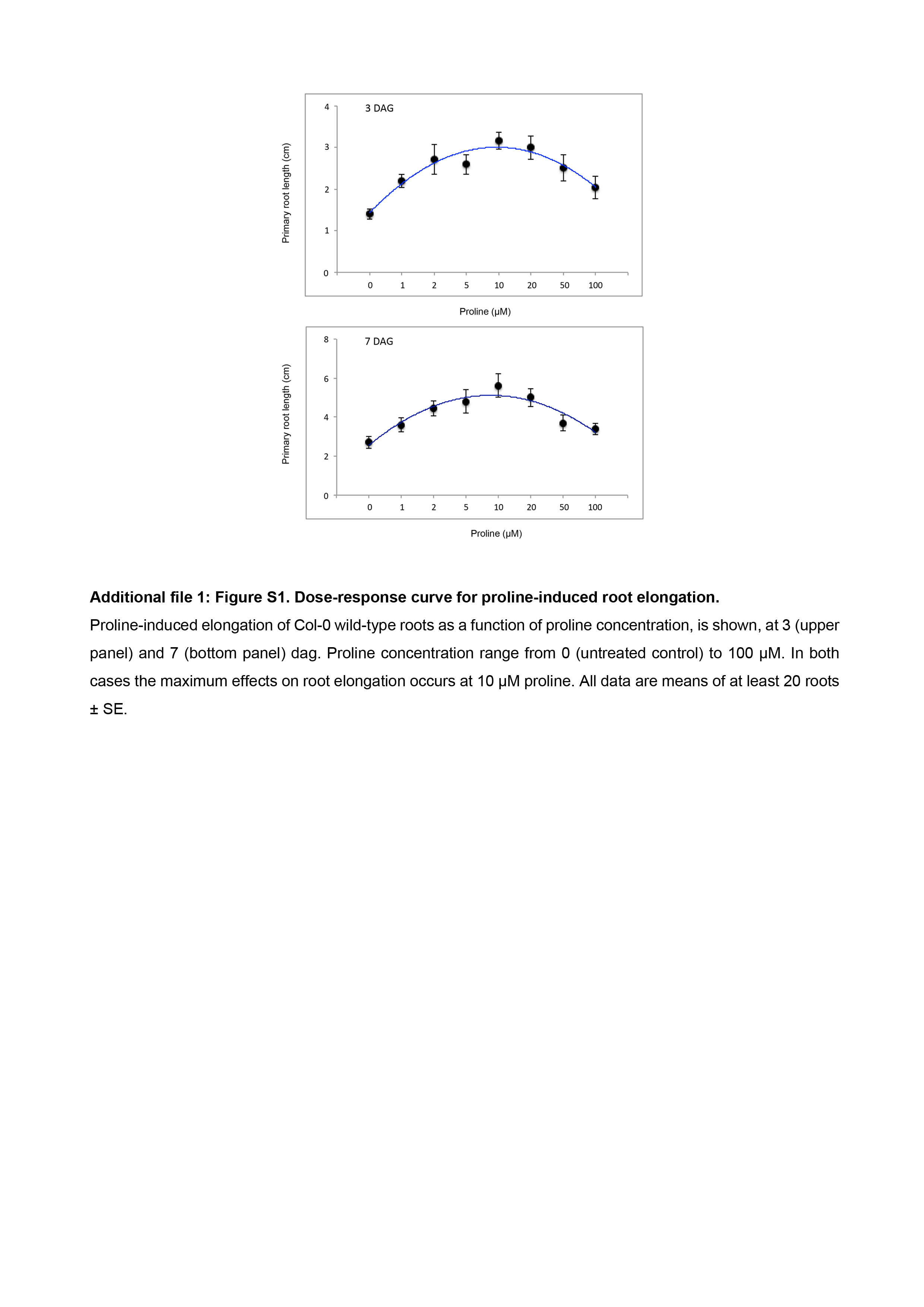

Supplement: Additional file 1: Figure S1. — Dose-response curve for proline-induced root elongation. Proline-induced elongation of Col-0 wild-type roots as a function of proline concentration, is shown, at 3 (upper panel) and 7 (bottom panel) dag. Proline concentration range from 0 (untreated control) to 100 μM. In both cases the maximum effects on root elongation occurs at 10 μM proline. All data are means of at least 20 roots ± SE. (PNG 194 kb) [file 12870_2015_637_MOESM1_ESM.png]

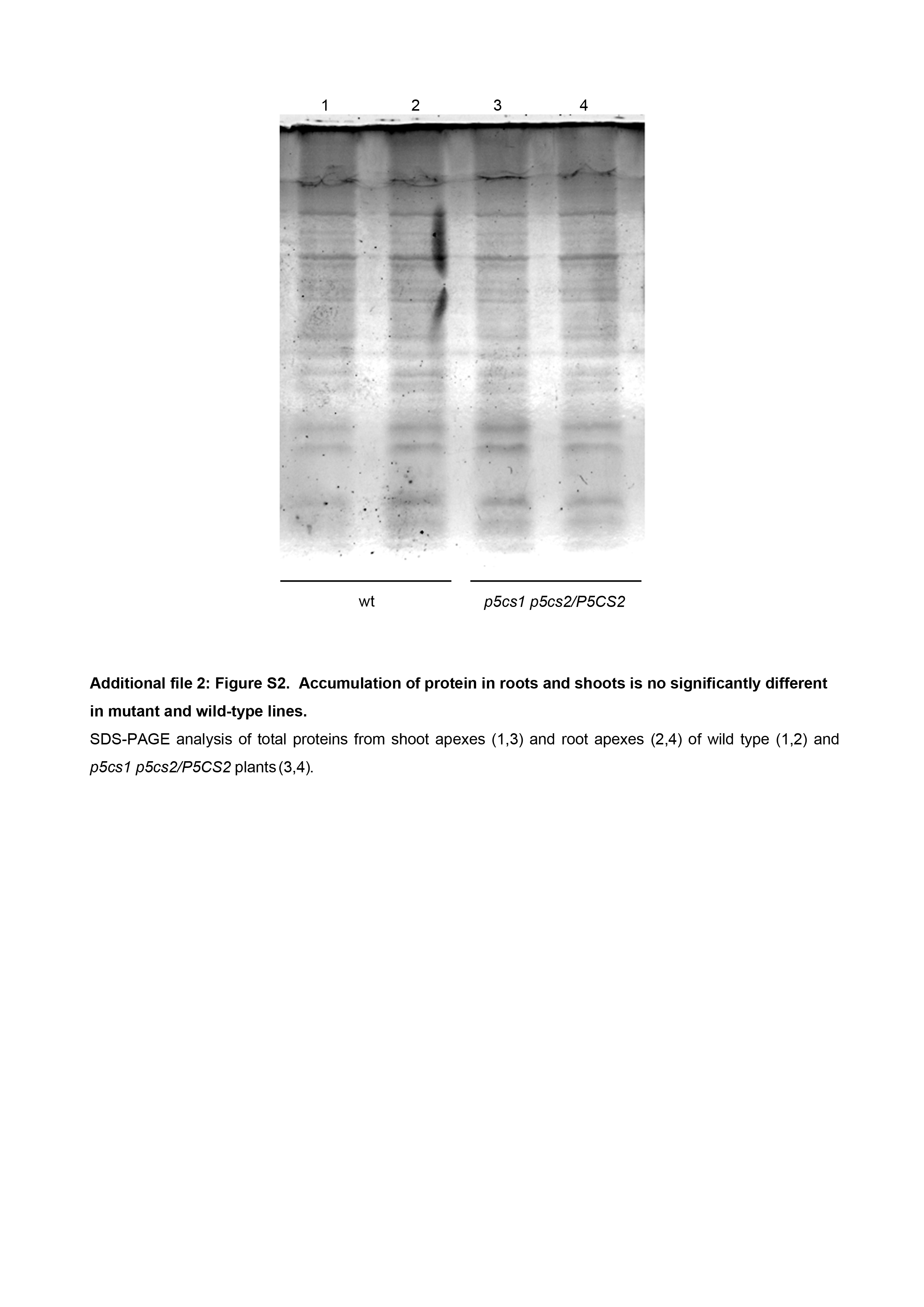

Supplement: Additional file 2: Figure S2. — Accumulation of protein in roots and shoots is no significantly different in mutant and wild-type lines. SDS-PAGE analysis of total proteins from shoot apexes (1,3) and root apexes (2,4) of wild type (1,2) and p5cs1 p5cs2/P5CS2 plants (3,4). (PNG 587 kb) [file 12870_2015_637_MOESM2_ESM.png]

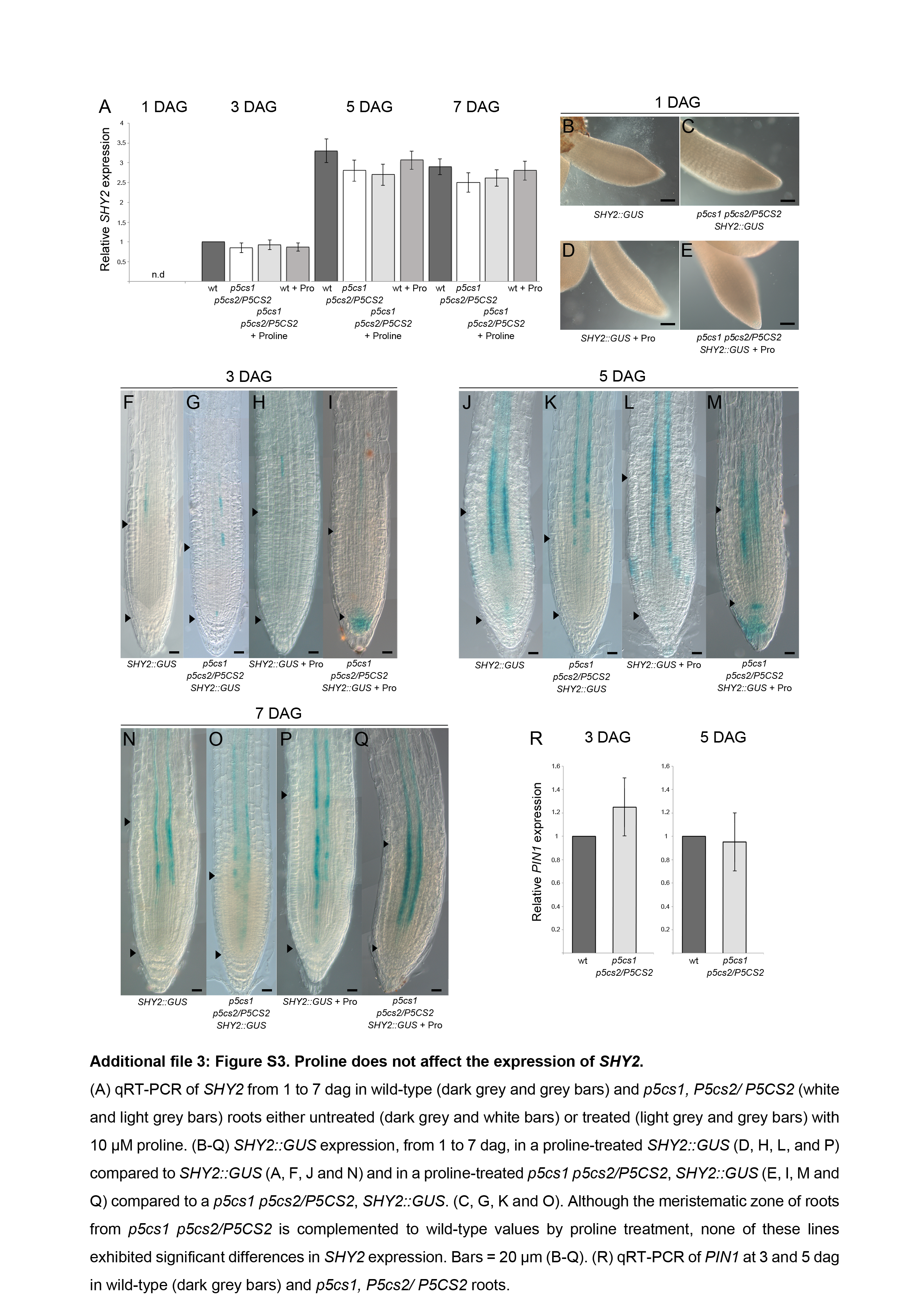

Supplement: Additional file 3: Figure S3. — Proline does not affect the expression of SHY2. (A) qRT-PCR of SHY2 from 1 to 7 dag in wild-type (dark grey and grey bars) and p5cs1, P5cs2/ P5CS2 (white and light grey bars) roots either untreated (dark grey and white bars) or treated (light grey and grey bars) with 10 μM proline. (B-Q) SHY2::GUS expression, from 1 to 7 dag, in a proline-treated SHY2::GUS (D, H, L, and P) compared to SHY2::GUS (A, F, J and N) and in a proline-treated p5cs1 p5cs2/P5CS2, SHY2::GUS (E, I, M and Q) compared to a p5cs1 p5cs2/P5CS2, SHY2::GUS. (C, G, K and O). Although the meristematic zone of roots from p5cs1 p5cs2/P5CS2 is complemented to wild-type values by proline treatment, none of these lines exhibited significant differences in SHY2 expression. Bars = 20 μm (B-Q). (R) qRT-PCR of PIN1 at 3 and 5 dag in wild-type (dark grey bars) and p5cs1, P5cs2/ P5CS2 roots. (PNG 3675 kb) [file 12870_2015_637_MOESM3_ESM.png]

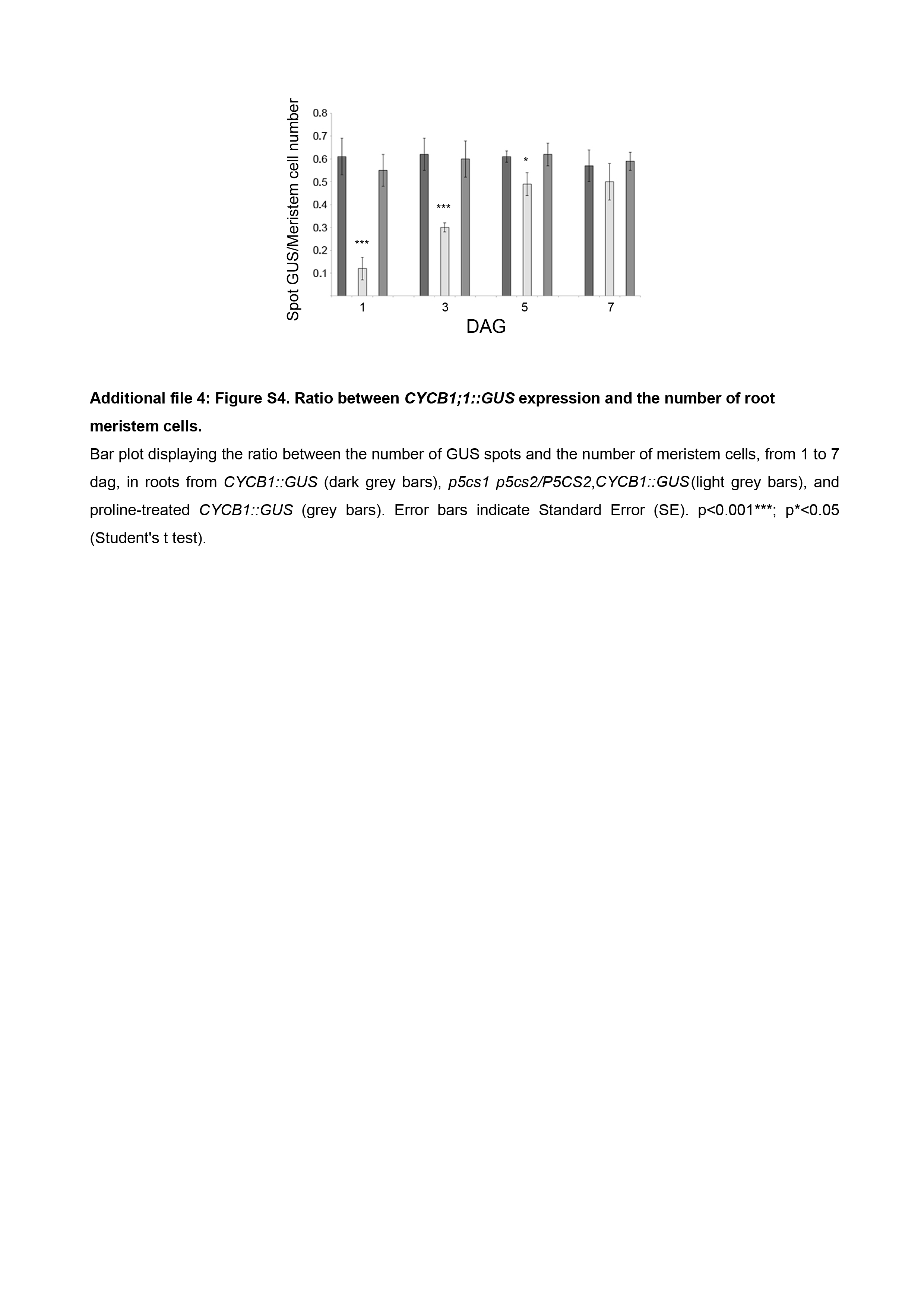

Supplement: Additional file 4: Figure S4. — Ratio between CYCB1;1::GUS expression and the number of root meristem cells. Bar plot displaying the ratio between the number of GUS spots and the number of meristem cells, from 1 to 7 dag, in roots from CYCB1::GUS (dark grey bars), p5cs1 p5cs2/P5CS2, CYCB1::GUS (light grey bars), and proline-treated CYCB1::GUS (grey bars). Error bars indicate Standard Error (SE). p < 0.001***; p* < 0.05 (Student’s t test). (PNG 129 kb) [file 12870_2015_637_MOESM4_ESM.png]

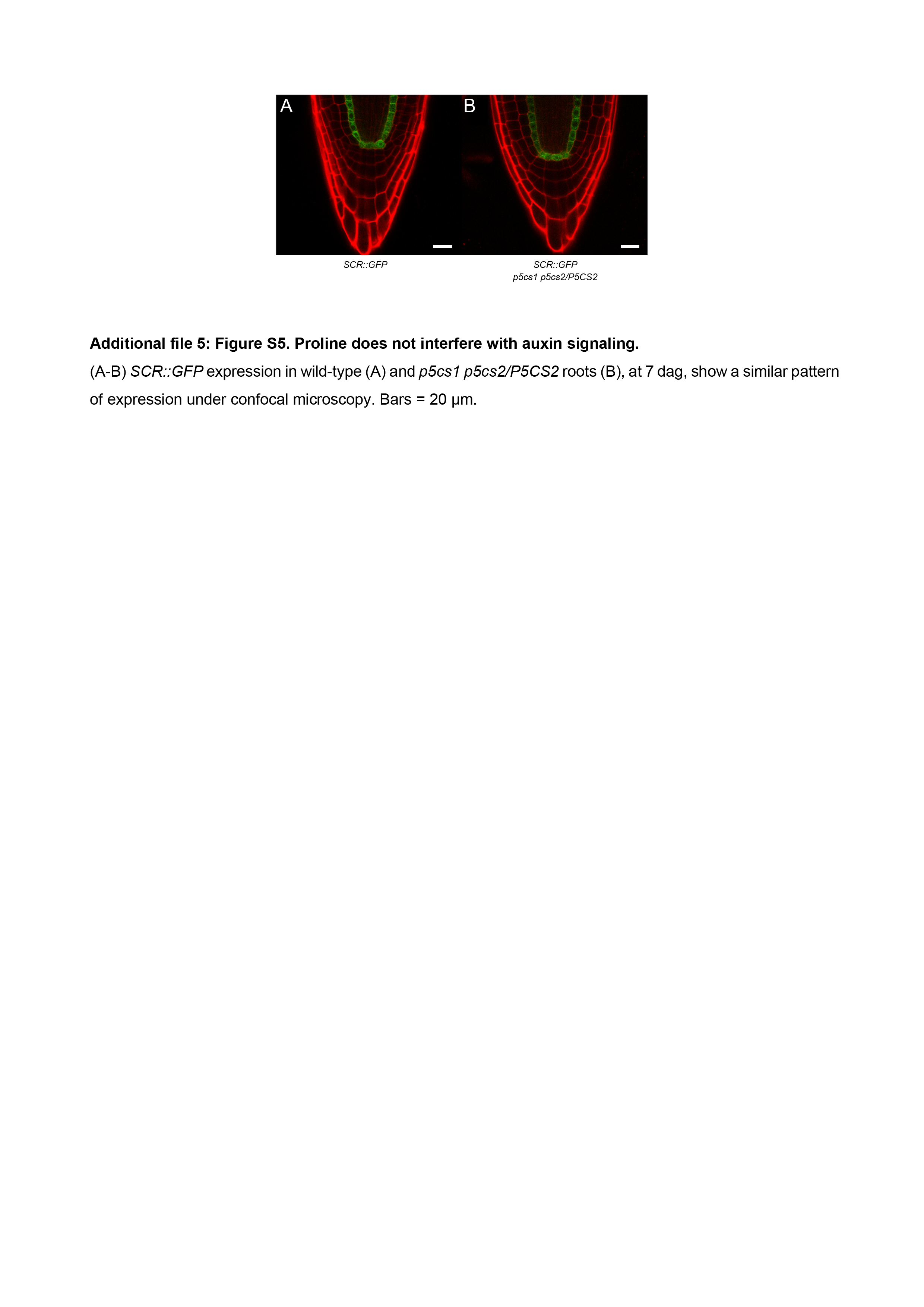

Supplement: Additional file 5: Figure S5. — Proline does not interfere with auxin signaling. (A-B) SCR::GFP expression in wild-type (A) and p5cs1 p5cs2/P5CS2 roots (B), at 7 dag, show a similar pattern of expression under confocal microscopy. Bars = 20 μm. (PNG 499 kb) [file 12870_2015_637_MOESM5_ESM.png]
